# An Implicit Measure of Associations with Mental Illness *versus* Physical Illness: Response Latency Decomposition and Stimuli Differential Functioning in Relation to IAT Order of Associative Conditions and Accuracy

**DOI:** 10.1371/journal.pone.0101911

**Published:** 2014-07-07

**Authors:** Stefania Mannarini, Marilisa Boffo

**Affiliations:** Department of Philosophy, Sociology, Education, and Applied Psychology, University of Padova, Padova, Italy; University of Udine, Italy

## Abstract

The present study aimed at the definition of a latent measurement dimension underlying an implicit measure of automatic associations between the concept of mental illness and the psychosocial and biogenetic causal explanatory attributes. To this end, an *Implicit Association Test* (IAT) assessing the association between the *Mental Illness* and *Physical Illness* target categories to the *Psychological* and *Biologic* attribute categories, representative of the causal explanation domains, was developed. The IAT presented 22 stimuli (words and pictures) to be categorized into the four categories. After 360 university students completed the IAT, a Many-Facet Rasch Measurement (MFRM) modelling approach was applied. The model specified a person latency parameter and a stimulus latency parameter. Two additional parameters were introduced to denote the order of presentation of the task associative conditions and the general response accuracy. Beyond the overall definition of the latent measurement dimension, the MFRM was also applied to disentangle the effect of the task block order and the general response accuracy on the stimuli response latency. Further, the MFRM allowed detecting any differential functioning of each stimulus in relation to both block ordering and accuracy. The results evidenced: a) the existence of a latency measurement dimension underlying the *Mental Illness* versus *Physical Illness* - *Implicit Association Test*; b) significant effects of block order and accuracy on the overall latency; c) a differential functioning of specific stimuli. The results of the present study can contribute to a better understanding of the functioning of an implicit measure of semantic associations with mental illness and give a first blueprint for the examination of relevant issues in the development of an IAT.

## Introduction

Research on the aetiology of psychiatric disorders has vastly expanded our knowledge on the genetic and neurobiological underpinning of mental illness [1]; on the other hand, the research on the aetiology of specific disorders, such as depression and more in general mood disorders, has pointed out the relevance of affective and social factors [2]. In the last decades, our understanding of mental illness has been improving (i.e., higher mental health literacy); however, the phenomenon of stigma towards mental illness still remains a heavy burden for affected people [3], [4]. Among other negative effects, stigmatizing attitudes and views of mental illness have been found to be related to lower quality of life, decreased willingness to seek treatment, treatment discontinuation and drop out (for a review, see [4]) [5], [6].

The *origin* of mental illness, i.e., the causes to which the condition is causally attributed, has been hypothesized to be one of the main components underlying stigmatizing processes towards mental illness [7], [8] and has been used as a promotional medium to overcome stigma in a number of public health programs aimed at combating discrimination (e.g., [9], [10]). These campaigns have been emphasizing the endorsement of biogenetic causal models of mental disorders by sponsoring a medical approach to mental illness (i.e., the “mental illness is an illness like any other” approach) and by explicitly portraying mental disorders as medical conditions that should be treated with medical treatments [11]–[13]. The promotion of biogenetic aetiological beliefs about mental illness has been deemed as a promising approach to reduce stigma, for this type of beliefs is connected to the perception of onset and offset controllability for the stigmatized condition. This perspective is well explained within the framework of attribution theory, which holds that the causal attribution of one’s behaviours leads to characteristic emotional, attitudinal, and behavioural responses towards the person in question [14]. The endorsement of biogenetic causes of mental illness is thus believed to reduce ascriptions of responsibility and guilt to the affected person, since such causes are beyond the individual control and may reverse the perception that people with a mental disorder are to blame for their troubles, with consequent less rejection in the social environment. Notwithstanding, campaigns sponsoring a biogenetic origin of mental illness did not fully produce the intended effects, with a mixed pattern of negative and positive results (e.g., higher levels of negative stigma but higher endorsement of professional mental health treatments and mental health literacy) [15]–[21]; whereas the consequences of the endorsement of a psychosocial aetiology of mental illness remained unchanged in the last 20 years [13].

The heterogeneity of findings pointed out the different theoretical accounts of mental illness stigma, which go beyond the scope of the present study, and the myriad of stigma measures, which is the starting point of the present study by calling for a standardization of measurement and for an exploration of instruments targeting more subtle processes in mental illness stigmatization.

A substantial line of research has indeed developed several instruments for the assessment of stereotypes, causal beliefs, cognitive representations, and affective reactions (e.g., negative attitudes and prejudice) related to mental illness [22]–[26] (for a review of measures of the different facets of mental illness stigma, see [27], [28]). It is rather evident that stigma entails a multifaceted and complex dimensional nature, which, in the last 20 years, prompted the design of a multitude of different instruments targeting one or more different aspect(s) of mental illness stigma at once.

So far, nearly all research on mental illness stigma has assessed individuals’ explicit attitudes, beliefs, and/or stereotypes by considering their voluntary and controllable features, as measured by self-report instruments [27]. However, certain attitudes can be discriminatory, such as those towards a minority out-group like mentally ill people, and can therefore increase the likelihood that social desirability concerns impact on self-reported views [29]. According to a dual-process account of explicit and implicit processes, implicit attitudes may be held regardless of whether an individual believes these to be true or false [30], [31]. Stigmatization processes involve then both automatic implicit responses as well as controlled deliberate responses [32], [34].

A relevant advance in research on stigma covers the investigation of the role of implicit processes in the expression of bias and the related development of measures assessing implicit cognitions and attitudes expressed outside the individual’s conscious control. Speeded reaction-time tasks such as the *Implicit Association Test* (IAT) [35] can measure automatic evaluative and semantic associations between two concepts and thus index, for instance, implicit attitudes (i.e., evaluative associations) and – like in the present study – beliefs towards mental disorders (i.e., semantic associations). Indirect measurement procedures such as the IAT can provide a behavioural measure of the strength of association among mental representations and they all rely on the assumption that the processing of a stimulus increases the accessibility of associated concepts [36].

A recent line of research has examined the value of including indirect assessments of implicit stigmatizing attitudes and stereotypes and of examining the role of stigma dual processes in both healthy and clinical samples, providing promising results about the differential functioning of negative attitudes and mental illness-related cognitive representations when measured at both the explicit and implicit level [37]–[43]. Teachman, Wilson, and Komarovskaya (2006) [37] showed that the general public, and even those diagnosed with a mental disease, presented a somewhat implicit and explicit bias against mentally ill people compared to physically ill people (see also [41]–[43]). Peris, Teachman, and Nosek (2008) further demonstrated the predicting value of implicit stigma assessment by finding that, although more implicit and explicit positive evaluations of mental illness were generally reported, more negative implicit attitudes toward mentally ill people predicted more over-diagnosis of clinical case vignettes than explicit attitudes in mental health professionals [39].

When considering the indirect assessment of mental illness-related causal beliefs, only one study addressed it specifically about depression compared to physical illness among psychology undergrads [40]. Implicit associations regarding the underlying psychological causes were found together with more negative evaluations. The effect of aetiological beliefs on mental illness negative evaluations emerged in another study by Rüsch and colleagues (2010) [42], where explicit mental illness biogenetic causal beliefs were associated to greater implicit self-guilt and explicit fear of mental illness amongst clinically diagnosed individuals.

The literature sketch provided so far encourages the experimentation of indirect measures for the assessment of more covertly expressed features of stigma, which can open an additional window on stigma by focusing on more automatic aspects of stigma towards mental illness. More precisely, the present work focuses on one of the elements hypothesized to be an antecedent or at least a component of stigma: the attribution of mental disorders to either biogenetic or psychosocial causal factors [7], [8]. The present research aimed at the development and psychometric investigation of a new implicit measure targeting the extent to which mental illness is automatically associated with the two causal explanatory domains.

The main purpose was to analyse the semantic automatic association between the concept of Mental Illness, relative to its natural and most obvious contrast category, i.e., Physical Illness [37], and the causal beliefs bipolar continuum represented by the two opposite Psychological and Biologic categories. To this aim, an IAT implicit measure was selected, which was based on the relative comparison between the association of *Mental Illness* and *Physical Illness* with the attribute dimension (*Psychological* versus *Biologic*).

The IAT was chosen for the following reasons:

- The relative nature of the concept of mental illness in comparison to the physical illness, in terms of salience and negative valence, prompted the use of a relative measure of implicit associations towards the two concepts (e.g., [37]);
- Within the corollary of indirect measures of implicit cognition, the IAT has been the most widely used and tested and is one of the most reliable [44]; these features suggested to use it as the starting point in the investigation of the measurement validity of an implicit measure of mental illness stigma processes.

According to the traditional IAT structure (see Table 1 in the Material and Methods section), stimuli pertaining to the *Mental Illness* and *Psychological* categories on one side and *Physical Illness* and *Biologic* categories on the other side, are hypothesized to be categorized faster when sharing the same response key (i.e., congruent associative task condition) than when sharing different response keys (i.e., incongruent associative task condition), pointing to a stronger association of target and attributive domain. It is here hypothesized that the individual associative network surrounding the concept of mental illness may influence the process of associating mental illness to the psychological or biological domain, following the automatic associations network account theorized by dual-process models of cognitions [30], [45].

**Table 1:** Task structure of the IAT for the assessment of the associations between *Mental Illness* and *Physical Illness* target categories and *Psychological* and *Biologic* attributes (critical blocks used in the MFRM analysis are emphasised).

Block	Task^a^	Stimuli	N° of trials	Left categorieslabels	Right categories labels
1	Target Practice	Words	20	Mental Illness	Physical Illness
2	Attribute Practice	Pictures	20	Psychological	Biologic
*3*	*Congruent combined practice*	*Words + Pictures*	*20*	*Mental Illness + Psychological*	*Physical Illness + Biologic*
*4*	*Congruent combined test*	*Words + Pictures*	*40*	*Mental Illness + Psychological*	*Physical Illness + Biologic*
5	Reversed target practice	Words	20	Physical Illness	Mental Illness
*6*	*Incongruent combined practice*	*Words + Pictures*	*20*	*Physical Illness + Psychological*	*Mental Illness + Biologic*
*7*	*Incongruent combined test*	*Words + Pictures*	*40*	*Physical Illness + Psychological*	*Mental Illness + Biologic*

a The block order was counterbalanced across participants with reversed target practice and incongruent combined blocks (practice and test) completed first in half of the participants.

From a measurement validity perspective, the analytical strategy of the present study involved the examination of the contributions of specific stimuli in triggering the enquired associations, by disentangling the functioning of the stimuli along the task in terms of speed of categorization. The analytical procedure guiding the present study is different from sorting the IAT trials into subsets and computing separate IAT effects for the target categories or for the single stimuli, which is not recommended in analysing IAT data [46]. Instead, the differential contribution of individual stimuli to the overall task performance was assessed. In particular, the main hypothesis embraces the different response speed with which people might categorize stimuli pertaining to the IAT key-categories presented in different combination formats along the task. This relative difference in the response latencies is interpreted as an *indirect* measure of the strength of the associative links between the concepts of interest. The examination of the functioning of each stimulus falls under the broader issue of the stimuli selection in the IAT, which is of crucial importance when devising such a measure for the possible misleading effects driven by the choice of inappropriate stimuli [46]–[47].

Furthermore, the response latency and accuracy, although combined together when responding to a stimulus, were also separately modelled to analyse their interaction in producing the final response outcome. The impact of the individual’s overall response accuracy in the IAT critical blocks was considered, since it might produce an effect on the stimuli response latency that goes beyond the enquired automatic associations (e.g., participants who are generally more accurate might have general longer reaction times or vice versa). An additional variable was considered in the analysis: the standard procedure of counterbalancing the IAT critical blocks. The reason for considering the order of presentation of the task blocks deals with the so-called ‘compatibility effect’, which refers to the facilitation effect of presenting the congruent block first on the task performance and which is one of the main criticisms raised towards the IAT [48]–[50]. The effect of this method-variable on the measure functioning was then explored.

The objective of going in depth into the functioning of the IAT measure here devised is to be considered as an attempt for a detailed analysis and decomposition of the ‘working mechanisms’ of an IAT. The methodology used for the analysis is inscribed in the family of Rasch models. The *Many-Facet Rasch Measurement* model (MFRM) [51] was applied to comply with the above-mentioned purposes and, first of all, to define the validity of the latent measurement dimension underlying the performance of the implicit measure.

The Rasch modelling approach has a long tradition in the development and psychometric analysis of psychological, educational, and medical assessment tools (e.g., [52]–[59]) and it has been used as a template that operationalises in a very flexible form the formal axioms of *additive conjoint measurement* [60], which underpin measurement and against which data collected from self-report measures may be tested for measurement validity [61]–[63]. Since the model defines measurement, data are fitted to the model to see if they meet the model expectations. This is opposite to the practice in statistical modelling where models are developed to best represent the data (e.g., Structural Equation Modelling). Fitting data to the Rasch model offers then an elegant approach to address several methodological key-aspects generally associated with scale development and construct validation, as well as providing a log-odds transformation of the ordinal raw scores.

Given the inner assessment features of implicit measures, including the IAT, the adoption of a Rasch modelling perspective seemed to be a possible answer to the question of whether it is possible to reach a deeper comprehension of the IAT measure and to run a first attempt to establish its measurement validity. The main idea underlying the application of a Rasch modelling perspective lies on the consideration of the stimuli categorization task required in the IAT as a variant of the item responding performance required when responding to traditional self-report measures. According to this conceptualization, IAT stimuli can thus be considered just like questionnaire items are traditionally conceived in a Rasch analysis, since in both cases respondents should reply according to the hypothesised underlying psychological process(es) and/or construct(s) [57]. Within this perspective, the methodological investigation of IAT stimuli in terms of measurement validity and reliability was then directly faced in a fashion that resembles the test development approach applied to traditional assessment measures and addressed within a latent trait modelling framework by applying the MFRM.

There are several advantages for using the MFRM, and more in general Rasch models, in the investigation of implicit associations and implicit measures:

- All Rasch models conform to the properties of stochastic independence, specific objectivity, linearity, and measurement unit (for a discussion, see [64]);
- The MFRM allows modelling, besides the traditional subject and item parameters, other variables, or *facets*, that might interfere and affect the outcome of a rating process (traditional self-report measures) or of a task (stimuli categorization), such as, in the case of the present study, the order of presentation of the task associative conditions and the general response accuracy [51];
- All model parameters, or facets, are located on the same latent continuous trait, allowing comparisons between their elements [65];
- All facets lie on a common latent dimension of categorization latency; the speed of categorization of the stimuli is expressed by interval measures characterized by a common measurement unit, which, if the data fit the model, maintains the same size over the entire continuum;
- As a consequence of the specific objectivity, the measures obtained by the model are sample-, stimulus-, condition-, and all other facet-free and can be compared with any other;
- Specific goodness-of-fit statistics assess the fit of the data to the model and are highly informative about the results interpretation of each single stimulus, participants, response accuracy, order of task blocks, or any other relevant variable in the model;
- The MFRM allows interaction analyses among different facet parameter estimates, to detect any differential functioning of any facet parameter estimate in relation to the other variables entered in the model. This feature is of great importance in so far as it provides a powerful tool to examine systematic patterns of deviations from the model expectations in the data, and to identify possible factors causing this patterns (e.g., the procedure of counterbalancing the blocks in speeded reaction time-based tasks).

A detailed description of the MFRM model applied in the present study appears further in the Material and Methods section.

## Materials and Methods

### Ethics Statement

In keeping with the local Institution Ethics Review Board regulation, the study was presented during regular lectures in the university course of Research Methodology in Psychology as an example of how to conduct scientific research in Psychology. Participants who refused to take part in the research did not incur any disadvantage in fulfilling the course requirements.

Written informed consent was obtained from all participants after the procedure had been fully explained and data anonymity and for research use only was guaranteed. Participants were told that they were totally free to withdraw from the study at any time. Responses were anonymised by assigning to participants an identification number before starting the study. Collected data were then aggregated and analysed at the group level.

### Participants

The study involved 360 undergraduate students of the University of Padua (75% of the approached students; mean age = 23.82 years, *SD* = 3.146).

### Materials and Procedure

#### The mental illness causal beliefs Implicit Association Test

The IAT [35] measures the association strength between pairs of target concepts and an attributive dimension and consists of a stimuli categorization task into super-ordinate categories. It is a relative measure, so the *Mental Illness* target category was compared with associations of *Physical Illness* on the bipolar dimension of psychosocial (*Psychological*) versus biogenetic (*Biologic*) causal explanations of mental illness. *Mental Illness* was contrasted to *Physical Illness* for two main reasons: first, mental illness is a negative concept given that it reflects illness, so physical illness seemed the most obvious comparison term for its salience and since it is akin to entail negative evaluations as mental illness (e.g., [37], [40]–[43]), though it is not as stigmatized as mental illness [37]. A second reason relied on the “mental illness is an illness like any other” approach, which has been promoting the conception of mental disorders as medical conditions to be treated with medical interventions, leading to the consideration of physical illness as the most effective contrasting category [12].

The IAT consists of two binary categorization tasks combined together so that the sorting task is compatible or incompatible with the to-be-measured associations. It implies the classification of words and/or pictures as quickly and accurately as possible into four categories differently labelled, by pressing either a left (E) or a right (I) key on the keyboard.

The IAT comprises seven blocks of trials (see Table 1): three single practice blocks of categorization of stimuli pertaining to either two target or two attribute categories, and four critical blocks (two practice combined blocks and two test combined blocks), which involve the simultaneous double categorization of stimuli pertaining to the target and attribute categories combined together on two response mappings presented on the top left and right sides of the screen.

The logic behind the IAT is that stimuli are classified more quickly during one critical block, when the target and attribute category pairing (e.g., *Mental Illness*/*Psychological* versus *Physical Illness*/*Biologic*) matches respondents’ automatic associations between the two concepts, versus the other block, where the target and attribute category pairing is mismatched (e.g., *Mental Illness*/*Biologic* versus *Physical Illness*/*Psychological*). Therefore, an individual who presents a stronger automatic association of psychosocial features and elements with mental illness is expected to respond more quickly when *Mental Illness* and *Psychological* categories are paired and contrasted to *Physical Illness* and *Biologic* pairing (compatible block), when compared to the reversed pairing (incompatible block).

The participants were successively presented with mental and physical disease words and pictures depicting psychosocial situations and objects pertaining to the biology and natural sciences realms. The stimuli had to be classified into the *Mental Illness, Physical Illness, Psychological,* and *Biologic* categories. In one of the two double categorization tasks (blocks 3 and 4), the two single classification tasks are combined in such a way that participants have to respond to *Mental Illness* words and *Psychological* pictures with one key (E), and to *Physical Illness* words and *Biologic* pictures with another key (I). In the other double categorization task (blocks 6 and 7), the target and attribute categories pairing is reversed.

The IAT was administered on a 15-inch personal computer in a controlled laboratory setting. Each block was preceded by a short instruction page reminding the exact key assignment and to be ready to correct the answer in case of mistake (a red cross appeared at the centre of the screen).

The presentation order of the combined tasks was counterbalanced across participants [46]. From now on, the term *Compatible* refers to the block order condition in which participants completed the congruent task first, whereas the term *Incompatible* refers to the reversed block order.

#### IAT stimuli

A set of stimuli for the four IAT categories was developed by selecting the most representative exemplars for each concept. Within each IAT block the stimuli were presented equally often in a random order across participants on a blank black background.

#### *Target categories stimuli*

For each target category five words were chosen from a set of 20 exemplars for both concepts. The selection of ten mental diseases and ten physical diseases followed the diagnostic categories of DSM-IV-TR and ICD10 and a Google search typing Italian words for different mental and physical diseases and picking up the most cited. The 20 words were then evaluated by a team of experts in mental health care and medicine on the following criteria: ease of categorization into the appropriate category, frequency and usage of the word in the daily language, and representativeness of the target concept. For the *Mental Illness* category the five selected words were the following: *depression*, *schizophrenia*, *psychopathy*, *paranoia*, and *hysteria*. For the *Physical Illness* category the five stimuli were *tumour*, *heart attack* (the Italian translation is a single word: *infarto*), *pneumonia*, *flu,* and *diabetes*.

#### *Attribute categories stimuli*

For each attribute, *Psychological* and *Biologic*, six pictures were chosen from a set of 15 cartoon images depicting common exemplars of the two concepts. The use of pictures instead of words was due to the difficulty in conveying aspects of biological and psychological semantic areas by means of words without ambiguity or misinterpretation. A Google search of pictures was carried out by using key words such as ‘relationship’, ‘psychological’, ‘social’, ‘psychology’, ‘biology’, ‘genetics’, and ‘natural sciences’ and looking for coloured and stylised cartoons. The final 12 pictures had to meet the following criteria: representativeness of the domain and pictorial features (e.g., clarity, similarity in clearness, colours, quality, low degree of ambiguity). For the *Psychological* category, the six pictures depicted several interpersonal relationships: a *mother/child relation*, a *grandparents/grandchildren relation*, a *work meeting*, *peers fighting*, a *romantic couple relation*, and a *family relation*. For the *Biologic* category, the six pictures depicted several objects pertaining to the area of natural sciences, biology, and chemistry: the image of a *cell* under the microscope, a *filament of DNA*, a coloured image of an *atom structure*, *test tubes* for clinical analysis, a *microscope*, and two *chromosomes*.

To improve the visual understanding of the pictures an instruction page at the beginning of the task informed the participants that the category *Psychological* referred to the individual experience and the relations between individuals and the surrounding environment, whereas the category *Biologic* referred to anything related to the organic and biological aspects of life.

### Data Pre-processing

Prior to the MFRM analysis, the IAT data were reduced and checked out for abnormal response patterns according to the standard IAT data cleaning procedure [66]. Only critical trials (blocks 3, 4, 6, and 7) data were used for the analyses. Latencies greater than 10000 ms were discarded from the dataset. No participants presented response latencies lower than 300 ms in 10% of the trials. Two participants were excluded from the dataset as their error rate was >25% across the entire task, resulting in a dataset of observations for 358 participants.

For each stimulus *i* the median value of its response latencies distribution in the pooled critical blocks was computed. The median statistic was chosen as a measure of the latency central tendency of participants’ responses to the stimuli due to the lower sensitivity to the distribution tails than the mean statistic. The distribution of the stimuli median latencies was successively discretised into a three-category rating scale according to two percentile values (33^rd^ and 66^th^) computed on the N participants×22 stimuli full data matrix to identify fast, medium, and slow response latency [67]. This discretization procedure complies with the Rasch modelling requirement of entering only discrete variables in the model.

Participants’ average response accuracy and Compatible and Incompatible order conditions were further added in the matrix. Similarly to the response latency data discretization procedure, the percentage distribution of correct responses in the pooled critical blocks was discretised into two categories according to its median value. Participants were then indexed by two levels of average response accuracy, Low (below the median value) or High (above the median value).

Given the acknowledged effect of the counterbalanced order of task blocks presentation on the IAT effect and task performance [48]–[50], participants’ responses in the Compatible/Incompatible block order conditions were compared in the MFRM analysis, to verify whether the counterbalancing procedure did affect the stimuli categorization task and contributed to the hypothesized measurement latent dimension. A binary variable sorting out participants’ assignment to the two block order conditions was then added in the data matrix.

Data collected and used for the analyses in the present study are available upon request.

### The Model

The MFRM [51] derives from the *Simple Logistic Model* (SLM) [68], which is the traditional and most basic Rasch model for the transformation of ordinal observations into interval measures. The SLM is meant for dichotomous data and expresses, according to a logistic distribution, the probability of a response *x* to a test, which can be correct (1) of incorrect (0), as an additive function of the ability of respondent *v* and difficulty of the item *i*, as expressed on the *logit* scale 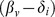 [68].

However, in the measurement contexts complex situations are more the rule than the exception, and other aspects may interfere with the person and item attributes, such as specific experimental, social, and personality attributes. Within the context of Rasch modelling mono-dimensionality and mathematical properties (for a review, see [62]), Linacre [51] developed an extension of the SLM, namely, the MFRM, which extends the analysis to more complex situations by including other sources of systematic variability (*facets*), in addition to respondents’ ability and item (or stimulus) difficulty, accounting for the likelihood of a response.

In the present study, besides the person (facet 1) and the stimuli parameters (facet 2), the parameters describing the facilitating effect of the block order (facet 3) and the general accuracy (facet 4) on response times were added in the model equation. An additional parameter accounting for the response latency rating scale 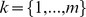 provided by the response latency distribution discretisation was embedded in the model. The MFRM model equation was then formally expressed as follows:
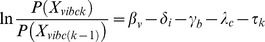



The MFRM Equation specifies the probability that a respondent *v* would respond to stimulus *i* in the task order setting *b* with a general accuracy level *c,* with a response speed *k* rather than 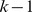; 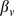 is the person *v* ability (categorization ability/speed) parameter, 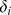 is the stimulus *i* difficulty (ease/speed of categorization) parameter, 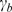 identifies the facilitation effect of the order *b* of presentation of the critical blocks on the categorization speed, 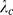 denotes the speed of categorization of respondent’s general level of accuracy *c*, and 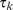 is the parameter for the step up to category *k* rather than 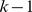 of the response latency rating scale.

The Rasch model parameters are additive, thus satisfying one of the requisites for interval measures, and are based on the transformation of scores into a *logit* scale, i.e., the logarithmic transformation of the probability of giving a particular response given certain conditions (e.g., participants’ ability, stimuli recognisability, difficulty of the block order, categorization speed at different levels of response accuracy). In the MFRM Equation, the *logit* of a certain response *k* can be seen as the dependent variable, whereas the various factors act as independent variables that influence (or control) the response.

All parameter estimates were positively scaled in the analyses, so that high positive values indicate fast responses, whereas negative measures indicate slow responses.

To evaluate the goodness-of-fit of the parameter estimates, the MFRM presents two fit statistics that show how much the data for each parameter adhere to the model requirements: the *mean square Outfit* and *mean square Infit* statistics. These statistics are calculated for each participant, each stimulus, and any other facet parameter and express the relationship between observed and model-derived expected responses, ranging from zero to infinity (for details, see [69]). A range of. 70–1.30 indicates a satisfactory fit of the observed data to the model requirements [64].

A Chi-square statistic – the *Fixed (all same) χ^2^* – is also provided for each facet and tests the hypothesis that the elements of a facet have the same *logit* in relation to the measurement error (*SE*). In other words, the Chi-square statistic helps to reject the null hypothesis that there is no group-level difference in the different elements composing a facet. For instance, a *Fixed (all same)* χ^2^ with an associated *p*-value lower than. 05 for the stimuli facet points to the presence of group-level differences among the stimuli, which are sorted out with different speeds. A conceptually similar index is the separation reliability (R), which indicates how well the elements of a facet are separated to reliably represent the facet and ranges from 0 to 1. It reflects an estimation of the relationfship between true scores and true variance: 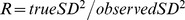, where *observedSD* is the standard deviation of the estimates (not corrected for measurement error).

Once estimated each facet measure, it is possible to compare different parameter estimates by standardizing their paired difference, which approximates to the Student’s *t* distribution, 
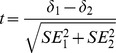
, with degrees of freedom (*df*) equal to the sum of the respective *df*s.

The MFRM gives the possibility to carry out the bias/interaction analysis, i.e., the analysis of the interactions between elements of different facets [70]. A bias can be due to any kind of interaction, such as differential item functioning, differential person functioning or differential functioning of any other facet, and is estimated from the residuals left over after estimating the parameters in the main analysis and tested for statistical significance by means of a *t* statistic. This feature allows identifying possible factors causing any systematic deviation from the model expectations in the data, such as the ordering condition the stimuli are presented in (i.e., Compatible or Incompatible). In particular, the *differential stimulus functioning* (DSF) analyses the interaction between elements of the facet stimuli and elements of other facets. For instance, the bias index involves introducing an interaction parameter between the facets into the model (e.g., for the stimuli ⊥ block order interaction). The *logit* of a stimulus *i* in the order condition *b* is computed by adding a bias smeasure to the overall speed of categorization of the same stimulus *i* if the response to the latter is faster in that order condition than overall and by subtracting it if the response to the stimulus is slower. The two biased stimulus measures are then subtracted and tested via the above-mentioned paired *t*-test.

The application of the MFRM analysis to the IAT data was operationalized as follows:

1. Verification of a common latent trait wherein the IAT stimuli parameter estimates and participants’ parameter estimates express their location on the latent dimension on a common measurement unit, which describe their speed of categorization;
2. Estimation of the other facets parameters (i.e., block order and general accuracy), which allows a decomposition of the overall response latency on the measurement dimension;
3. Analysis of the effect of task block order on the stimuli functioning across the critical blocks (bias/interaction analysis);
4. Analysis of the effect of the two-way interaction of task block order and general accuracy on the stimuli functioning;

The analyses were performed using FACETS 3.60.0 software [71].

## Results

### The Person Facet

The mean value of the person parameter estimates (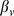) was equal to .03 (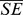 = .41, range = [−5.26, 5.61], *SD* = 1.51), with satisfactory Infit and Outfit statistics in the range. 70–1.30. The *Fixed Chi-Square* evidenced that participants presented significantly different categorization speed estimates on the latent dimension (χ^2^_(357)_ = 3044, *p*<.001, R = .91).

### The Stimulus Facet

Each stimulus parameter estimate (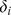) presented satisfactory Infit and Outfit statistics in the range. 70–1.30, signalling that they are measuring a common latent trait. Stimuli estimates of categorization speed were satisfactorily distributed along the latent dimension, indicating that they can well represent different levels of difficulty in the speed of categorization of the task (χ^2^_(21)_ = 1721.8, *p<*.001, R = .99). In Table 2 parameter estimates, Standard Errors, and Infit and Outfit statistics for each stimulus are presented.

**Table 2:** Stimulus format, latency parameter estimate (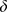), Standard Error (*SE*), and Infit and Outfit statistics for each MIPI-IAT stimulus.

Stimulus	Stimulus Format	δ	*SE*	Infit	Outfit
1. Depression	Word	.99	.09	.88	.82
2. Schizophrenia	Word	.53	.09	.86	.86
3. Psychopathy	Word	.44	.09	.86	.83
4. Paranoia	Word	.78	.09	.95	.94
5. Hysteria	Word	.93	.09	.90	.84
6. Tumour	Word	.46	.09	.91	.87
7. Heart attack	Word	.91	.09	.91	.93
8. Pneumonia	Word	1.42	.10	.84	.92
9. Flu	Word	1.05	.09	.93	.95
10. Diabetes	Word	1.11	.09	.97	.98
11. Mother/child relation	Picture	−.58	.09	1.02	.98
12. Grandparents/grandchildren relation	Picture	−.72	.09	1.13	1.12
13. Work meeting	Picture	−.54	.09	1.09	1.22
14. Peers fighting	Picture	−.68	.09	1.18	1.23
15. Romantic couple relation	Picture	−.76	.09	1.04	1.08
16. Family relations	Picture	−.61	.09	1.00	.96
17. Cell	Picture	−.60	.09	1.22	1.24
18. DNA filament	Picture	−.98	.09	1.08	1.14
19. Atom structure	Picture	−.72	.09	1.12	1.08
20. Test-tubes	Picture	−.87	.09	1.00	.97
21. Microscope	Picture	−.76	.09	1.06	1.06
22. Chromosomes	Picture	−.79	.09	1.13	1.08

The 22 stimuli were then considered as indicators of the latent dimension underlying the IAT, representing the speed of association of the *Mental Illness* and *Physical Illness* categories with the *Psychological* and *Biologic* domains (*Mental Illness versus Physical Illness-Implicit Association Test* - MIPI-IAT). The stimuli from 1 to 10 (words for the target categories *Mental Illness* and *Physical Illness*) showed positive parameter estimates in the range [.43–1.42] (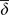 = .86), whereas the remaining stimuli from 11 to 22 (pictures for the attribute categories) presented negative estimates in the range [−.98, −.54] (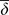 = −.72). This finding indicates that in general words were categorized faster, whereas pictures required a longer time to be categorized, independently from the category they belonged to. Among the attribute stimuli, the slowest stimuli were objects pertaining to the area of natural sciences, such as the *test tubes*, the *DNA filament*, and the *chromosomes*.

The cross-tabulation of the types of stimuli (word vs. pictures) by the four IAT categories (*Mental Illness* vs. *Physical Illness* and *Psychological* vs. *Biologic*) can provide further information about the interpretation of the stimuli parameter estimates. By considering the word stimuli, *Physical Illness* stimuli (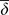 = .99), were categorized faster when compared to the *Mental Illness* stimuli (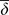 = .73). In other words, the processes needed to recognize and sort mental illness exemplars (e.g., depression, paranoia, and hysteria) were slower than the categorization processes of physical diseases, such as tumour, flu, and diabetes. *Psychological* pictures presented a mean latency estimate equal to −.65, which was slightly greater than the mean latency estimate of *Biologic* visual stimuli (−.78). This means that the stimuli referring to the psychological evaluative category were categorized slightly faster.

### The Block Order Facet

The Compatible (C) and Incompatible (I) order of critical blocks resulted to be located on the latent continuum and were categorized with different speeds (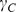 = .17, *SE* = .03; 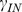 = −.17, *SE* = .03; χ^2^_(1)_ = 81.2, *p*<.001, R = .99), with Infit and Outfit statistics in the range. 70–1.30. The two parameter values suggested that in the Compatible order of presentation the sorting task was performed quicker than in the Incompatible condition. In other words, at the group level people who completed the congruent critical blocks first replied faster than people who completed the critical blocks in the reversed order.

### The Response Accuracy Facet

The High (H) and Low (L) general accuracy levels were associated to different speeds of categorization on the latent dimension (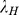 = −.20, *SE* = .03, 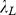 = .20, *SE* = .03; χ^2^_(1)_ = 107.2, *p*<.001, R = .99). At the group level, low general response accuracy was associated to quicker responses.

Both accuracy parameter estimates presented satisfactory Infit and Outfit statistics in the range. 70–1.30.

### The Stimuli Differential Functioning

#### The effect of the IAT block order on stimuli response latency

The bias/interaction analysis for stimuli and order of presentation of the critical blocks showed several DSFs. The response latency parameter estimate was significantly smaller in the Compatible order condition for stimuli 12 (*grandparents/grandchildren relation*, *p* = .026) and 14 (*peer fighting*, *p* = .005), compared to the Incompatible block order. Conversely, the response latency measure was smaller in the Incompatible condition for stimuli 7 (*heart attack*, *p* = .005) and 21 (*microscope*, *p* = .05). These results showed that four out of the 22 stimuli were differently categorized in the two block order conditions: two pictures pertaining to the *Psychological* category and two stimuli pertaining to the *Physical Illness* and the Biologic category, respectively. No statistically significant evidence was found for the remaining stimuli, showing that although participants who completed the congruent critical blocks first replied on average faster than people who completed the critical blocks in the reversed order, the comparison between Compatible and Incompatible order conditions on each stimulus response latency showed only a few affected stimuli.

#### The interaction effect of response accuracy and block order on stimuli response latency

Accuracy levels and block order were cross-tabulated for each stimulus, obtaining twenty-two 2
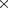
2 tables. In each table a stimulus latency estimate for each interaction was present: a) Low accuracy/Compatible, b) Low accuracy/Incompatible, c) High accuracy/Compatible, and d) High accuracy/Incompatible. To sum up the results of the interaction analysis, a 2×2 summary table was created for each IAT category and a latency estimate mean value for each accuracy by block order interaction was computed throughout the 22 tables previously created (see Table 3).

**Table 3:** Accuracy by block order interaction effects on stimuli parameter estimates for each MIPI-IAT target and attribute category: mean latency parameter estimates (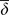).

	Mental Illness target stimuli			Psychological attribute stimuli		
	*Block order*			*Block order*		
*Accuracy*	Compatible	Incompatible	*Mean*	Compatible	Incompatible	*Mean*
Low	.998	.964	.*981*	−.308	−.648	−.*478*
High	.452	.504	.*478*	−.762	−.923	−.*843*
*Mean*	.*725*	.*734*		−.*535*	−.*785*	
	**Physical Illness target stimuli**			**Biologic attribute stimuli**		
	***Block order***			***Block order***		
***Accuracy***	**Compatible**	**Incompatible**	***Mean***	**Compatible**	**Incompatible**	**Mean**
Low	1.056	1.266	*1.161*	−.687	−.458	−.573
High	.722	.922	.*822*	−.959	−1.013	−.986
*Mean*	.*889*	*1.094*		−.*823*	−.*736*	

In the top left part of Table 3, which describes *Mental Illness* stimuli, the accuracy marginal mean values showed that the fastest responses (.981) were given in the Low accuracy condition, whereas the slowest responses (.452) were found for the interaction High accuracy/Compatible. In the top left part of Table 3, where *Psychological* stimuli are summarized, the accuracy marginal mean values showed that the fastest responses (−.478) were given in the Low accuracy performance, whereas the slowest responses (−.923) occurred in the combination of Incompatible block order and High accuracy. Also in the bottom left part of Table 3, which considers *Physical Illness* stimuli, faster responses (1.161) were associated to Low accuracy and, similarly to *Mental Illness* stimuli (−.573), the slowest responses (.722) were found in the High accuracy/Compatible cross-tabulation. In the bottom right part of Table 3, the results concerning *Biologic* attribute stimuli were similar to the *Psychological* ones, i.e., the accuracy marginal mean values showed that the fastest responses (−.573) were related to the Low accuracy, whereas the slowest responses (−1.013) were associated to the interaction High accuracy/Incompatible. The block order marginal mean values did not differ significantly across the four sub-tables, except for the Psychological stimuli, where responses were clearly faster (−.535) in the Compatible order.

To analyse the interaction effect of general accuracy and block order on each stimulus latency estimate, a single, comprehensive index (*L*) was devised, which combines the effect of the two variables. The parameter estimates for response accuracy and block order were cross-tabulated for each stimulus. Twenty-two 2×2 tables (see Table 3) were then obtained, where a latency parameter estimate for each interaction between the accuracy levels, Low (*l*) and High (*h*), and the block orders, Compatible (*c*) and Incompatible (*i*), was computed. The *L* measure was then obtained by combining the four interaction parameter estimates 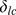, 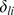, 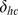, and 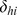 previously estimated:
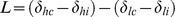



In order to define the standardized form of *L,* two standard values were then calculated:

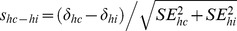
 was the standard value contrasting Compatible (*c*) and Incompatible (*i*) block order at a High (*h*) level of accuracy;

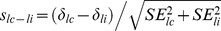
 was the standard value for the same contrast at the Low (*l*) level of accuracy.

The standardized *S(L)* index was then computed by subtracting the two standard values, 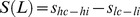), with:

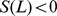
 when 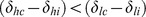, i.e., the effect of the block order is smaller at a high accuracy level;

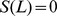
 when 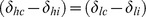, i.e., the effect is equal at both accuracy levels;

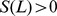
 when 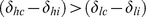, i.e., the effect s larger at a high accuracy level.

The standardized latency indices *S(L)* for the 22 stimuli are presented in Table 4. On average, in 45.4% of stimuli the block order effect was smaller (negative sign) when response accuracy was high. In other words, for these stimuli a higher accuracy was related to a decreased influence of the counterbalanced order of presentation of the critical blocks on the response latencies. Of these stimuli, the majority belonged to the *Mental Illness* and *Psychological* categories. The remaining 55.6% of stimuli showed a positive *S(L)* index value, indicating that the blocks order effect was higher even when the accuracy level was also high. This means that despite of the presence of a general high level of accuracy, the order of presentation of the critical blocks affected the categorization of these stimuli. In this case most of the stimuli belonged to the *Biologic* category.

**Table 4:** Stimulus, latency parameter estimate (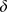), standardized contrast values for block order by general accuracy levels, and standardized latency index *S(L)*.

		Compatible/Incompatible block order contrast: *Standard value*					Compatible/Incompatible block order contrast *Standard Value*		
Stimulus	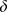	High Accuracy 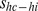	Low Accuracy 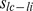	*S(L)*	Stimulus	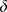	High Accuracy 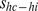	Low Accuracy 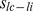	*S(L)*
1^a^	.99	−.38	1.12	−1.50	12^b^	−.72	.37	2.83**	−2.46**
2^a^	.53	−1.27	−1.05	−.22	13^b^	−.54	.27	1.24	.97
3^a^	.44	.95	−.21	1.16	14^b^	−.68	2.70**	1.36	1.34
4^a^	.78	.24	1.24	−1.00	15^b^	−.76	.77	−.45	1.22
5^a^	.93	−.61	−.49	−.12	16^b^	−.61	−.14	2.10*	−2.24**
6^a^	.46	−1.29	−.31	−.98	17^b^	−.60	.99	−.31	1.30
7^a^	.91	−1.30	−2.80**	1.5	18^b^	−.98	−.12	−.64	.52
8^a^	1.42	−.51	−1.16	.65	19^b^	−.72	−.15	1.08	−1.23
9^a^	1.05	−.52	.99	−1.51	20^b^	−.87	.74	−2.08*	2.82**
10^a^	1.11	−.31	−.42	.11	21^b^	−.76	−.94	−1.87*	.93
11^b^	−.58	−.27	1.14	−1.41	22^b^	−.79	.78	−1.61	2.39**

a Words; ^b^Pictures; *p≤.05; **p<.01.

When testing the statistical significance of the *S(L)* index, Table 4 shows statistically significant results for stimuli *grandparents/grandchildren relation* (*S(L)* = −2.46, *p*<.01), *family relation* (*S(L)* = −2.24, *p*<.01), *test tubes* (*S(L)* = 2.82, *p*<.01), and *chromosomes* (*S(L)* = 2.39, *p*<.01). For these stimuli the effect of the ordering conditions on the latency measures was clearly different when distinguishing the two levels of response accuracy.

Within a measure validation perspective, this result demonstrated that 18 out of the 22 stimuli did not present any differential stimulus functioning, i.e., they did not evidence any significant bias related to the individual response accuracy and the IAT block order conditions.

## Discussion

The present study aimed at the development of a latent measurement dimension representing an implicit measure of the semantic associations of mental illness with the psychological and biological domains, to evaluate the extent to which the concept of mental illness, relative to physical illness, is automatically associated to the two causal explanatory domains. To this aim, an IAT was designed. The adoption of a latent trait modelling approach in the form of the MFRM model pursued the psychometric investigation of the hypothesised latent dimension. The model identified the targeted measurement dimension wherein the IAT stimuli are located according to their latency parameter estimates, which describe the ease of categorization in terms of speed into the IAT categories they belonged to. Furthermore, the response latency was decomposed into the additive combination of several facets, i.e., the person’s general categorization speed, the stimuli easiness of categorization, the facilitating effect of the IAT method-variable of block ordering, and the general response accuracy in the critical blocks.

### The Definition of the IAT Dimension

The 22 stimuli showed a satisfactory fit to the model requirements and identified the latent dimension underlying the performance of the *Mental Illness versus Physical Illness-Implicit Association Test* (MIPI-IAT). The ten word-stimuli were categorized faster than the 12 picture-stimuli, meaning that it was easier for the respondents to categorize both *Mental Illness* and *Physical* Illness target categories than *Psychological* and *Biologic* attribute categories. This might be due to several reasons. The actual type of stimuli used, words versus pictures, may have a different effect on the task performance and on the response latency – since words emerged to be processed quicker that pictures – as already pointed out in the discussion on implicit measurement [46]. This result might be also due to the stimuli belongingness to either the target categories (word-stimuli) or the attribute categories (picture-stimuli), pointing to the possibility that the effect arises at the nominal level of super-ordinate categories, particularly the target concepts, which are more easily sorted out than the attribute categories. This interpretation could also appeal to a possible asymmetry in salience between the target and the attribute categories.

However, although targets were categorized quicker than attributes, when considering the target stimuli only, it was also evidenced that *Physical Illness* stimuli were recognized faster than *Mental Illness* stimuli. This result may deal with the higher familiarity in daily life with physical health problems, but also with the fact that mental illness exemplars might differ in valence (and also in salience) when compared to physical diseases. However, these hypotheses need to be further tested, since no familiarity and/or valence measure was provided to the participants.

On the other hand, when considering the picture-stimuli only, it emerged that pictures depicting the *Psychological* category were categorized more easily (i.e., faster) than stimuli depicting the *Biologic* category. Also this result might be related to the higher familiarity with daily, social relationship-related contents, such as the mother-child or the grandparents-grandchildren relations, or to the difference in valence, since psychosocial aspects might as well entails a positive valence and therefore being categorised quicker than *Biologic* stimuli. The above-mentioned limitation applies in this case as well, since participants have not been asked to evaluate familiarity and valence of the pictures presented in the task.

Nonetheless, it could be also likely that the *Psychological* semantic category has been used by the participants as the salient dimension to perform the categorization task in the critical blocks, since when it first appeared in combination with the *Mental Illness* target category the task was easier and performed quicker than when it was first presented in combination with the *Physical Illness* target category. This hypothesis can encompass two possible accounts: a first one pointing to the hypothesised automatic association of mental illness to the psychological domain and physical illness to the biologic one. In this case, being presented first with a mismatched pairing could slow down responses and make the task more difficult, as evidenced by the Rasch measure of the Incompatible ordering. A second potential account for the difference in the categorization speed of the attributes and target categories could be related to a task-recoding strategy applied by the participants during the performance of the task [49]: participants could have re-coded the task from the classification of four elements into a pooled binary classification of physical illness-related cues on one side and psychological cues on the other side, by focusing on elements of these two super-ordinate categories and actively pairing the stimuli along another salient dimension available at the time.

Beyond the addition of a control measure of salience and/or valence, to probe these possible accounts of the difference in the categories classification a feasible strategy could be the use of a *Recoding-Free IAT* (RFIAT) [72], a *Single Category IAT* (SCIAT) [73], or a *Single Attribute IAT* (SAIAT) [74] for the assessment of the distinct, absolute associations towards each category (e.g., *Mental Illness*/*Psychological* versus *Mental Illness*/*Biologic*).

### The Contribution of the IAT Block Order and General Accuracy to the Stimuli Response Latency

Considering the response accuracy, the results showed the effect on the speed of categorization of generally performing the task more or less accurately, with a low accuracy associated to faster reaction times.

When the hypothesised congruent pairing (*Mental Illness/Psychological* versus *Physical Illness/Biologic*) was presented first, the sorting task resulted to be more efficient and faster than when the incongruent pairing was the first critical block (*Mental Illness/Biologic* versus *Physical Illness/Psychological*). This result is consistent with the literature on the ‘compatibility effect’, which evidenced how the discrimination task tends to be easier and quicker, with a consequent larger IAT effect, in the Compatible block order [48]–[50]. One of the acknowledged explanations for this order effect entails the assumption that presenting the congruent task first facilitates the emergence of the enquired automatic associations, thus impacting on the ease to which the automatic activation of associative links to the targeted concepts is triggered by the combined sorting task. The MFRM results evidenced how the order effect seems to impact directly upon the categorization speed and mostly on a few stimuli, which behave differently according to the order condition they are presented in. Response speed was significantly higher in the Compatible order condition for stimuli *grandparent/grandchildren relation* and *peer fighting*, whereas speed was higher in the Incompatible condition for stimuli *heart attack* and *microscope*. These four stimuli then presented a differential functioning related to the counterbalanced order of the critical blocks, most probably triggering the associate ‘compatibility effect’.

The analysis of the interaction effect of response accuracy by block order on the stimuli latency Rasch measures showed that in the Compatible ordering a high accuracy was combined to slower responses, in particular to word-stimuli pertaining to the target categories. When the stimuli pertained to the attribute categories, the opposite phenomenon occurred, namely, highly accurate and slow responses were observed in the Incompatible order condition. Once again, this result evidences the different categorization speed of the two pairs of categories after partitioning the ‘compatibility effect’ and the general level of accuracy of participants from the overall response latencies. However, we would be cautious from drawing the conclusion that a task-recoding strategy has been applied or that this difference is due to the activation of the targeted automatic associations between the targeted concepts. The first account needs to be further tested and not all possible response attractors have been considered in the model. Notwithstanding, these results highlight the importance for the development of categorization tasks of both the choice of the target categories under investigation and the different qualities and encoding paths of stimuli (words vs. pictures), since their categorization performance may result quite different for different reasons, as previously evidenced in the univariate analysis of the stimuli parameter estimates.

To further summarize the findings about the interaction effect of accuracy by block order on the stimuli latency, a new latency (*L*) index was proposed, which easily describes the order effect and participants’ general level of accuracy at the same time and directly elucidates in one shot the stimulus differential functioning resulting from the combination of the two facets. The standardized *S(L)* index evidenced that the combination of the block order and accuracy levels significantly affected only four out of the 22 stimuli. This means that 18 of the 22 stimuli were invariant across conditions and individual accuracy, suggesting that in large part the stimuli functioning was equal independently of task performance and IAT method variable.

To better explore and deepen the properties and possible applications of the latency *(L)* index, further analyses with different IAT versions and datasets are recommended.

## Conclusion

In summary, the MIPI-IAT devised in this study is represented by a latent measurement dimension composed of 22 stimuli. One of the main results claimed that word-stimuli for the *Mental Illness* category, such as schizophrenia, depression, or hysteria, required more time to be categorized than *Physical Illness* stimuli, such as pneumonia, flu, or diabetes. On the other hand, *Psychological* stimuli depicting psychosocial situations were recognized faster than stimuli depicting *Biologic* cues. A possible implication of this result focuses on the assessment utility of the MIPI-IAT, i.e., when people were asked to discriminate mental diseases exemplars, the implicit measure is able, at least to some extent, to catch the occurrence of uncontrolled and automatic underlying mechanisms that may interfere with participants’ response speed, including the higher familiarity with psychosocial representations when compared to objects pertaining to the natural and biological sciences realms, but also of possible task-recoding strategies in the performance of the task and, last but not least, of the enquired associative links between the targeted concepts and the attribute categories.

The subsequent step in the investigation of the implicit associations between mental illness and the explanatory realms of psychosocial and biogenetic causal attribution might be to separately test the potential contribution of each above-mentioned mechanism by, for instance, comparing on the same latent measurement dimension identified by the MFRM the performance of an absolute implicit measure (e.g., STIAT and SAIAT) and a structurally modified version of the IAT (e.g. RFIAT) using the same stimuli.

From a methodological perspective, the present study evidenced the usefulness of considering the specific contributions of stimuli and categories in the performance of an IAT. The MFRM model proved to be a suitable and flexible tool to devise a measurement latent dimension underlying the MIPI-IAT. The model represents a rigorous frame of reference in which estimating and comparing the speed of categorization of the stimuli. By allowing the analysis of differential stimulus functioning, the model unravelled the contribution of each stimulus to the overall IAT measure. Moreover, it allowed the decomposition of the stimuli response latency into the different facets that can interact one to each other in producing a response, such as the block order and the general accuracy.

Indeed, the Compatible order evidenced a pattern of fast and less accurate responses, suggesting a trade-off between speed and accuracy when the automatic association between *Mental Illness* and *Psychological* is activated first. Conversely, in the Incompatible order participants are slower and more careful, given the presentation of the incongruent block first (*Mental Illness/Biologic*), which is more difficult and in contrast with hypothesized implicit association between *Mental Illness* and the *Psychological* domain.

In order to provide further validity to the measurement properties of the MIPI-IAT, additional studies are recommended to better clarify the relations between target and attribute categories by also considering the type and variety of stimuli to be categorized. Further, the IAT was administered to a group of Psychology undergraduate students at the end of their university career, which could have contributed to the complex pattern of results. The respondents’ considerable mental health literacy and strong psychological background are arguable to have influenced the associative network in which the concept of mental illness resides. In order to refine the predictive properties and sensitivity of the measure, such analyses should be replicated with a laymen group and the MIPI-IAT should be compared with other related implicit and explicit measures to better define its theoretical, predictive, and practical aspects.

In conclusion, the MFRM analysis evidenced the MIPI-IAT to be a promising tool for the exploration of the stigmatizing processes towards mental illness. The use of the implicit measure here devised can be useful and potentially insightful, firstly to verify whether semantic associations to mental illness are related to more explicitly expressed prejudicial attitudes, and secondly to shed light on the theoretical status of the (implicit and explicit) components of stigma.
